# A randomized study protocol of microendoscopic versus open discectomy in treatment of lumbar disc herniation

**DOI:** 10.1097/MD.0000000000021361

**Published:** 2020-07-31

**Authors:** Yunlong Zhou, Zhiqiang Liu, Fei Lei, Kan Xie, Xufeng Jia

**Affiliations:** a Department of Orthopaedics, The People's Hospital of Leshan; b Department of Orthopedics, the Affiliated Hospital of Southwest Medical University; c Department of Orthopedics, The People's Hospital of Jianyang City, Sichuan Province, China.

**Keywords:** lumbar disk herniation, open discectomy, microendoscopic discectomy, protocol

## Abstract

**Background:**

Lumbar disk herniation (LDH) is one of the main causes of discogenic low back pain. However, the evidence comparing different approaches for discectomy has lacked definitive conclusions, with conflicting results regarding the benefit of minimally invasive versus open techniques for LDH. We are now conducting a randomized controlled trial to figure out whether or not microendoscopic discectomy yields better clinical outcomes and causes less surgical trauma than open surgery.

**Methods:**

This prospective, randomized, single-blind, controlled, superiority clinical trial was approved by the institutional review board in the People's Hospital of Jianyang City. The conduct of this study followed the Declaration of Helsinki principles and the reporting of this study adhered to the Consolidated Standards of Reporting Trials guidelines for randomized controlled trials. Subjects were randomized into 2 groups as follows: open surgery and microendoscopic group. The outcomes included pain score, functional outcome, satisfaction rate, radiological outcomes, and complications. The statistical analyses in this study were performed using the Statistical Package for the Social Sciences 20.0 software. *P* < .05 was accepted as statistically significant.

**Results:**

The hypothesis was that the open technique would achieve similar clinical outcomes as compared to the microendoscopic technique in LDH.

**Trial registration:**

This study protocol was registered in Research Registry (researchregistry5708).

## 1 Introduction

Lumbar disk herniation (LDH), one of the most common conditions for which patients visit the Department of Orthopedics, always carries a series of signs and symptoms. It is one of the main causes of discogenic low back pain and reported to affect 60% to 80% of people during their lifetime.^[1–3]^ Lumbosacral radiculopathy caused by the bulge of the nucleus pulposus and the secondary inflammatory reaction is the most challenging problem. Surgical intervention is required in patients whose symptoms fail to improve with conservative treatment.^[4–6]^

There are 2 main surgical modalities for intervertebral disc surgery: microendoscopic and open discectomy. Although various surgical options exist for lumbar disc herniation patients who do not respond to conservative treatment, open discectomy still remains a standard method.^[7,8]^ The traditional discectomy through laminotomy and microdiscectomy has obtained satisfactory results, but most experienced spine surgeons now prefer use of minimally invasive procedures that cause less trauma and lead to faster rehabilitation, such as percutaneous transforaminal endoscopic discectomy and microendoscopic discectomy, which are widely performed in treating LDH and achieve satisfactory clinical outcomes.^[9–12]^ However, some patients complain of persistent or recurrent radiating pain after minimally invasive discectomy, which can be accompanied by recurrence of the disc herniation.^[13]^

Previously, several observational studies have also tried to compare the efficacy and safety of these 2 procedures. However, the evidence comparing different approaches for discectomy has lacked definitive conclusions, with conflicting results regarding the benefit of minimally invasive versus open techniques for LDH.^[14–18]^ We are now conducting a randomized controlled trial to figure out whether or not microendoscopic discectomy yields better clinical outcomes and causes less surgical trauma than open surgery. The hypothesis was that the open technique would achieve similar clinical outcomes as compared to the microendoscopic technique in LDH.

## 2 Materials and methods

### 2.1 Study Design

**Figure 1:**
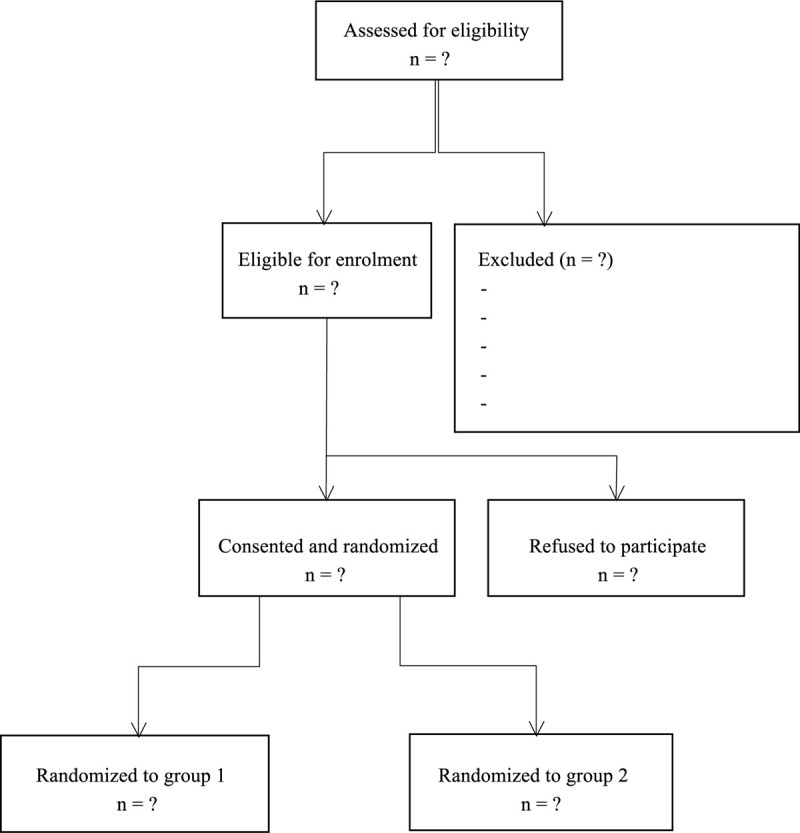
Flow of patients through the trial.

This prospective, randomized, single-blind, controlled, superiority clinical trial was registered in Research Registry (researchregistry5708) and approved by the institutional review board in the People's Hospital of Jianyang City (YNJY06940021). The conduct of this study followed the Declaration of Helsinki principles and the reporting of this study adhered to the Consolidated Standards of Reporting Trials guidelines for randomized controlled trials. The flowchart of this trial is shown in Figure 1.

### 2.2 Participants

The inclusion criteria for patients in this study included: age 20 to 90 years; persistent radicular pain lasting for >6 to 8 weeks; disc herniation confirmed by MRI single-level herniation; adjacent bisegmental herniation; desiccated disc with body root; entrapment/lateral canal stenosis; unilateral herniation was larger than one-third of the spinal canal diameter with concomitant lateral recess stenosis or “equestration.” Exclusion criteria were: <2 level disc herniation; cauda equina syndrome; spondylolytic or degenerative spondylolisthesis; spinal canal stenosis; pregnancy; severe somatic or psychiatric illness.

### 2.3 Randomization

Randomization was done by a secretary using a computer-generated randomization list (Research randomizer, www.randomizer.org) in a 1:1 ratio with 20 numbers in each block. Every participant received a consecutive study number from 1 to 69 and received the treatment assigned according to the randomization list. All clinical personnel and outcome assessors were blinded to the intervention. The randomization key was first broken when all enrolled patients had completed the study. After discharge, the participant's personal information was eliminated from the study number and was therefore not traceable back to the patients.

### 2.4 Techniques

#### 2.4.1 Open surgery group

Without the use of operating microscope, 8 to 10 cm medline skin incision was centered over the affected level after fluoroscopic verification. Using cutting diathermy dorso-lumbar fascia was incised, then stripping the paraspinal muscles off the spinous processes and lamina was performed until the facet joints laterally, then muscles were retracted laterally using a self-retaining retractor. Using Kerrison's Rongeur we did hemilaminectomy depending on the preoperative planning then locating and removal of the extruded or the sequestrated disc material.

#### 2.4.2 Microendoscopic group

An 18-mm tubular retractor was inserted over the sequential dilators that were inserted over a guide wire directed to superior lamina of the desired level then the rigid endoscope was inserted into the tubular retractor. Partial flavectomy was performed after laminotomy in which we limit the excision of the Ligamentum flavum enough to see the lateral edge of the dural sac and the traversing nerve root then retract them both medially with their covering of ligamentum flavum, then perform discectomy. In almost all cases we found the large extrusion directly under the ligamentum flavum. We could search for caudal sequestration by placing the endoscope cephalic and with the help of angled ball probe we could retrieve part or all of the sequestration by sweeping under the dural sac or posterior longitudinal ligament or searching into intervertebral foramen and lateral recess then we pull it out with the help a rongeur.

### 2.5 Clinical Outcome Measures

The outcomes included pain score, functional outcome, satisfaction rate, radiological outcomes, and complications. Pain score and functional outcomes were assessed by using a visual analogue scale (VAS, 0-10) and the Oswestry Disability Index (0-100%), respectively. Subjective surgical satisfaction rate (%) was assessed by asking the patient, “How satisfied were you with this operation?” Pre- and postoperative data were assessed by clinical charts and operation records. Radiographs were assessed preoperatively and at the 2-year follow-up.

### 2.6 Sample Size Calculation

The sample size calculation was based on a pilot study that we conducted on eightteen patients (whose data were not included in the present study). In this previous study, the mean difference and standard deviation of the VAS scores after the operation at 1 year between the open and microendoscopic groups were 0.52 and 0.21, respectively. From this, it was determined that 60 subjects would be required to reach an α value of 0.05 and a power of 90%. It was estimated that the attrition rate due to canceled surgery or reasons of late patient ineligibility could be up to 20% and, therefore, to account for this, the final sample size selected was n = 140 (70 per group).

### 2.7 Statistical analysis

The statistical analyses in this study were performed using the Statistical Package for the Social Sciences 20.0 software. Continuous variables were presented in the form of mean ± standard deviation or error. The Kolmogorov-Smirnov normality test was used to assess continuous variables. Group comparisons on the variables that showed normal distribution were performed using one-way analysis of variance. Mann–Whitney *U* variance analysis was used for discrete numerical variables that did not show normal distribution. Relationships between the categorical variables were determined by preparing crosstabs and using the χ^2^ test. *P* < 0.05 was accepted as statistically significant.

## 3 Discussion

Low back pain has become one of the most serious public health problems, with a lifetime prevalence as high as 84% and the prevalence of chronic low back pain is about 23%, with 11% to 12% of the population being disabled by low back pain.^[4,7]^ To date, the factors that eventually cause pathological progression have not been determined. However, along with recent economic development, living, environmental, and working conditions have substantially changed in China. Lumbar disc herniation is one of the most common spinal degenerative disorders leading to LBP associated with radiculopathy. On the other hand, some studies found that disc herniation was actually common in asymptomatic people as well. Inflammatory response has been acknowledged to be important in the process of disc degeneration and may play an important role in pain generation.^[12–14]^

We are now conducting a randomized controlled trial to figure out whether or not microendoscopic discectomy yields better clinical outcomes and causes less surgical trauma than open surgery. The hypothesis was that the open technique would achieve similar clinical outcomes as compared to the microendoscopic technique in LDH. The main limitation of the present study was the inability to blind both the participants and the physicians to comparisons between peripheral nerve blockade and periarticular injection. This lack of blindness may have introduced some risk of bias from both the patients and the physicians. The outcome assessments from the adjudicators and all the statistical analyses were conducted in a blinded manner.

## Author contributions

**Conceptualization:** Yunlong Zhou, Zhiqiang Liu.

**Data curation:** Yunlong Zhou, Zhiqiang Liu.

**Formal analysis:** Yunlong Zhou, Zhiqiang Liu.

**Funding acquisition:** Xufeng Jia.

**Investigation:** Yunlong Zhou, Zhiqiang Liu.

**Methodology:** Fei Lei.

**Resources:** Kan Xie.

**Software:** Kan Xie.

**Supervision:** Xufeng Jia.

**Validation:** Xiaoyan Qin.

**Visualization:** Kan Xie.

**Writing – original draft:** Yunlong Zhou, Zhiqiang Liu.

**Writing – review & editing:** Xufeng Jia.
